# The future of metabolomics in ELIXIR

**DOI:** 10.12688/f1000research.12342.2

**Published:** 2017-10-30

**Authors:** Merlijn van Rijswijk, Charlie Beirnaert, Christophe Caron, Marta Cascante, Victoria Dominguez, Warwick B. Dunn, Timothy M. D. Ebbels, Franck Giacomoni, Alejandra Gonzalez-Beltran, Thomas Hankemeier, Kenneth Haug, Jose L. Izquierdo-Garcia, Rafael C. Jimenez, Fabien Jourdan, Namrata Kale, Maria I. Klapa, Oliver Kohlbacher, Kairi Koort, Kim Kultima, Gildas Le Corguillé, Pablo Moreno, Nicholas K. Moschonas, Steffen Neumann, Claire O’Donovan, Martin Reczko, Philippe Rocca-Serra, Antonio Rosato, Reza M. Salek, Susanna-Assunta Sansone, Venkata Satagopam, Daniel Schober, Ruth Shimmo, Rachel A. Spicer, Ola Spjuth, Etienne A. Thévenot, Mark R. Viant, Ralf J. M. Weber, Egon L. Willighagen, Gianluigi Zanetti, Christoph Steinbeck

**Affiliations:** 1 ELIXIR-NL, Dutch Techcentre for Life Sciences, Utrecht, 3503 RM, Netherlands; 2 Netherlands Metabolomics Center, Leiden, 2333 CC, Netherlands; 3 ADReM, Department of Mathematics and Computer Science, University of Antwerp, Antwerp, 2020, Belgium; 4 ELIXIR-FR, French Institute of Bioinformatics, Gif-sur-Yvette, F-91198, France; 5 Department of Biochemistry and Molecular Biomedicine, Faculty of Biology, Universitat de Barcelona, Barcelona, 08028, Spain; 6 School of Biosciences, Phenome Centre Birmingham and Birmingham Metabolomics Training Centre, University of Birmingham, Birmingham, B15 2TT, UK; 7 Computational and Systems Medicine, Department of Surgery and Cancer, Imperial College London, London, SW7 2AZ, UK; 8 INRA, UNH, Human Nutrition Unit, PFEM, Metabolism Exploration Platform, MetaboHUB-Clermont, Clermont Auvergne University, Clermont-Ferrand, F-63000, France; 9 Oxford e-Research Centre, Engineering Science Department, University of Oxford, Oxford, OX1 3QG, UK; 10 Leiden Academic Centre for Drug Research, Leiden University, Leiden, 2300 RA, Netherlands; 11 European Molecular Biology Laboratory, European Bioinformatics Institute (EMBL-EBI), Cambridge, CB10 1SD, UK; 12 Centro Nacional Investigaciones Cardiovasculares, Madrid, 28029, Spain; 13 CIBER de Enfermedades Respiratorias, Madrid, 28029 , Spain; 14 ELIXIR Hub, Cambridge, CB10 1SD, UK; 15 Toxalim, UMR 1331, Université de Toulouse, Toulouse, F-31300, France; 16 Metabolic Engineering and Systems Biology Laboratory, Institute of Chemical Engineering Sciences, Foundation for Research & Technology – Hellas (FORTH/ICE-HT), Patras, GR-26504, Greece; 17 Biomolecular Interactions, Max Planck Institute for Developmental Biology, Tübingen, 72076, Germany; 18 Department of Computer Science, University of Tübingen, Tübingen, 72076, Germany; 19 Center for Bioinformatics, University of Tübingen, Tübingen, 72076, Germany; 20 The Centre of Excellence in Neural and Behavioural Sciences, Tallinn, Tallinn, 10120, Estonia; 21 School of Natural Sciences and Health, Tallinn University, 10120, 10120, Estonia; 22 Department of Medical Sciences, Uppsala University, Uppsala, 752 36, Sweden; 23 UPMC, CNRS, FR2424, ABiMS, Station Biologique, Roscoff, F-29680, France; 24 Department of General Biology, School of Medicine, University of Patras, Patras, GR-26504, Greece; 25 Department of Stress and Developmental Biology, Leibniz Institute of Plant Biochemistry, Halle, 06120, Germany; 26 BSRC “Alexander Fleming”, Athens, GR-16672, Greece; 27 Magnetic Resonance Center, Interuniversity Consortium for Magnetic Resonance on MetalloProteins, University of Florence, Florence, 50121, Italy; 28 Luxembourg Centre For Systems Biomedicine (LCSB), University of Luxembourg, Belvaux, L-4367, Luxembourg; 29 Department of Pharmaceutical Biosciences, Uppsala University, Uppsala, 752 36, Sweden; 30 CEA, LIST, Laboratory for Data Analysis and Systems’ Intelligence, MetaboHUB, Gif-sur-Yvette, F-91191, France; 31 Department of Bioinformatics - BiGCaT, NUTRIM, Maastricht University, Maastricht, NL-6200, Netherlands; 32 CRS4, Data Intensive Computing Group, Ed.1 POLARIS, Pula, 09010, Italy; 33 Friedrich-Schiller-University, Jena, 07743, Germany

**Keywords:** metabolomics, databases, bioinformatics infrastructure, data standards, computational workflows, cloud computing, training, multi-omics approaches

## Abstract

Metabolomics, the youngest of the major omics technologies, is supported by an active community of researchers and infrastructure developers across Europe. To coordinate and focus efforts around infrastructure building for metabolomics within Europe, a workshop on the “Future of metabolomics in ELIXIR” was organised at Frankfurt Airport in Germany. This one-day strategic workshop involved representatives of ELIXIR Nodes, members of the PhenoMeNal consortium developing an e-infrastructure that supports workflow-based metabolomics analysis pipelines, and experts from the international metabolomics community. The workshop established *metabolite identification* as the critical area, where a maximal impact of computational metabolomics and data management on other fields could be achieved. In particular, the existing four ELIXIR Use Cases, where the metabolomics community - both industry and academia - would benefit most, and which could be exhaustively mapped onto the current five ELIXIR Platforms were discussed. This opinion article is a call for support for a new ELIXIR metabolomics Use Case, which aligns with and complements the existing and planned ELIXIR Platforms and Use Cases.

## Introduction

Metabolomics aims to provide novel insights into the biochemistry of organisms by characterising the presence and concentrations of low molecular weight compounds from biological samples. It measures both endogenous (produced within an organism) and exogenous (those introduced from the environment including food components and drugs) metabolites. The primary analytical tools for such high-throughput data collection are mass spectrometry (MS), often preceded by chromatographic or electrophoretic separation technologies, and nuclear magnetic resonance spectroscopy (NMR). These technologies produce relatively large and complex data sets that require bioinformaticians, cheminformaticians, biostatisticians and computer scientists to develop and apply a wide range of algorithms, software tools, repositories and computational resources to process, analyse, report and store the data and metadata.

**Figure 1.**
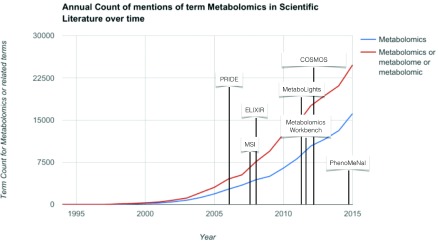
Annual count of mentions of the term ‘metabolomics’ (and related terms) in the literature over time, annotated with important milestones in data sharing and standards in metabolomics. Source: Data from Google Scholar.

The field celebrated its coming of age in 2016 ^1^ and progressed primarily through developments in analytical and computational tools, from which biomedical discoveries followed. As shown in Figure 1, the term ‘metabolomics’ is still gaining momentum and the global market for metabolomics was valued at $5.9 billion in 2014 and was expected to reach $12.5 billion by 2020, with a compound annual growth rate (CAGR) of 13.0% ( https://goo.gl/yXTiJD). The future is bright for the application of metabolomics in academic and industrial laboratories, scientific instrument companies, government laboratories and contract research organisations. Yet, several challenges remain. Discussions amongst both independent metabolomics experts, and those within ELIXIR ( http://elixir-europe.org/), culminated at the recent workshop the “Future of metabolomics in ELIXIR”. This opinion article summarises the interactions in the workshop and its outcomes.

ELIXIR coordinates bioinformatics resources across its member states and help researchers to find, analyse, and exchange biological data. It is a distributed infrastructure with a single Hub based in Hinxton, United Kingdom, and an increasing number of Nodes located throughout Europe. As of July 2017, ELIXIR has 20 national Nodes, with European Bioinformatics Institute (EMBL-EBI; co-located with the Hub), working as a separate Node.

### Workshop “The future of metabolomics in ELIXIR”

The workshop was organised at the Airport Conference Center on April 25 ^th^ 2017 in Frankfurt (Germany). The invitation to the workshop was widely advertised through the newsletter of the Metabolomics Society ( http://metabolomicssociety.org), the PhenoMeNal (Phenome and Metabolome aNalysis) project website ( http://phenomenal-h2020.eu) and ELIXIR dissemination channels, including ELIXIR Technical Coordinators, Heads of Node mailing lists and the ELIXIR newsletter ( https://goo.gl/KVUb8y). The invitation was also extended to the partners of the MetaStar consortium ( http://meta-star.eu, the H2020 Metabolomics Starting Community), and interested members of the metabolomics community.

The workshop included 35 participants from across 10 countries, representing the ELIXIR Nodes including Belgium, EMBL-EBI, Estonia, France, Germany, Greece, Italy, Luxembourg, The Netherlands, Spain, The United Kingdom, and the ELIXIR hub. The objective of the meeting was to identify the principal challenges within this field and prioritise actions, in particular those within the scope and mission of ELIXIR. The workshop showcased flash presentations on the national metabolomics activities in the participating Nodes and ideas and requirements for future developments of ELIXIR metabolomics activities. An overview of ELIXIR Use Cases and Platforms was also presented by Rafael Jiménez (ELIXIR Chief Technology Officer), representing the ELIXIR Hub. The presentations were followed by discussions on the needs and challenges present in the metabolomics community. The following challenges were identified:

1. Minimum information standards and early data capture
2. Global spectral databases
3. Tools and standards registries
4. Compound identifier mapping
5. Omics data integration
6. Metabolite identification

These challenges are described in more detail in the following paragraphs.

## Identification of challenges

### Minimum information standards and early data capture

In 2007 the Metabolomics Standards Initiative (MSI) ^2, 3^ published a set of seminal papers with recommendations on minimal reporting standards for a number of aspects in metabolomics, summarized in 4. This work built on earlier efforts by the Standard Metabolic Reporting Structure initiative ^5^ and the Architecture for Metabolomics consortium (ArMet) ^6^. Although critical, the MSI work served only as a starting point. Since then, several initiatives have been set up to address elements of standardisation, particularly on data standards, building on the work of the MSI. The COSMOS project (COordination Of Standards In MetabOlomicS, http://www.cosmos-fp7.eu) ^7^, coordinated data standards efforts amongst database providers, ontologists, software engineers and instrument vendors working towards open access data standardization and agreements. A Metabolomics Society *Data Standards Task Group* was subsequently established to foster and coordinate efforts in enabling efficient storage, compression, terminological annotation, exchange and verification of information within metabolomics datasets. However, no significant and coordinated effort has since been attempted on minimum reporting itself: there is now widespread agreement that the early work of the MSI needs to be taken up in a new initiative, linked with related activities such as the HUPO-PSI ( http://www.psidev.info), and be ‘completed’. Indeed, the lack of standardised and validated reporting in the field of metabolomics has now become a significant hindrance for data reuse and the translation of this technology into regulated areas of science. For example, one conclusion from a European workshop on regulatory toxicology stated that “there are a number of R&D needs, including a database to support metabolomics, standardisation, validation and reporting formats” ( https://goo.gl/EiuTfM), and progress is now underway ^8^ ( https://goo.gl/HapKhz). The multiple task groups of the international Metabolomics Society have objectives to educate and reform reporting standards including the Metabolite identification and QA/QC task groups.

### Global spectral databases

In contrast to genomics, there is a lack of metabolite databases with sufficient depth and breadth covering entire metabolomes. There are no databases capable of providing a near comprehensive snapshot of the molecular diversity available.

While a number of databases around the globe store and serve spectral data, there is no open and comprehensive resource targeted at the needs of metabolomics. The public repository MassBank ( http://www.massbank.jp/en/database.html), which stores mass spectral data, is actively developed and used by members of the metabolomics community. However, it is designed to hold only reference spectra and not experimental raw data. For NMR, a few and sparsely populated repositories provide raw data for individual metabolites, such as the metabolomics collection in BioMagResBank ( http://www.bmrb.wisc.edu/www/resources.shtml) ^9^.

Efficient metabolite identification requires several fundamental resources and developments, which mandate concerted efforts across research groups and countries in Europe and worldwide. In the field, two main types of resources exist:

1. Databases of measurements: Resources such as the Human Metabolome Database (HMDB, http://www.hmdb.ca) ^10^, and the Yeast Metabolome Database (YMDB, http://www.ymdb.ca) ^10, 11^ store MS and NMR measurements of known compounds but also collect and curate published results with the aim to establish specific organism reference metabolomes.
2. Genome-based metabolic reconstruction databases: These databases are built based on genome annotation, mostly of enzymes and their association to known reactions, and thus may not cover the entire metabolome since some genes that are enzymes have no enzymatic function associated and some functions may not yet be known. Normally they do not include spectral data, but they are valuable in associating expected metabolites to defined species. Examples include KEGG ^12^, BioCyc ^13^ and Recon ^14^, which is a human genome-scale metabolic reconstruction.

Metabolite databases such as the commercial Reaxys database ( https://www.reaxys.com), open-access Chemical Entities of Biological Interest (ChEBI, https://www.ebi.ac.uk/chebi/ ^15^), and KNApSAcK ^16^ ( http://kanaya.naist.jp/KNApSAcK/) contain data on species-metabolite associations, besides those mentioned in the Genome-based metabolic reconstruction databases category.

One important consideration in this arena is that different confidence levels can be associated with structural identification of metabolites ^17^, and standardized annotation schemes for such evidence descriptions are currently emerging ^18^. The highest confidence level can only be observed when instrumental data for an authentic chemical standard is matched to the data for the biological samples. However, authentic chemical standards are not available for many metabolites and therefore bioinformatics and chemoinformatics, including through ELIXIR supported resources, are essential to solve this community-wide hurdle.

Recently, as one strategy to accelerate the identification of metabolites in biological systems, a task group within the International Metabolomics Society has promoted the idea of characterising model organism metabolomes ^19, 20^. The philosophy of this task group is to leverage upon the critical mass of knowledge and activity that already exists for model organisms, linking to these other efforts and exploiting resources such as sequenced genomes to predict metabolism (as targets for experimental investigation) using genome-wide metabolic reconstructions or metabolic pathway databases KEGG ^12^, BioCyc ^13^, and WikiPathways ^21^. The task group has set a grand challenge for the community, to identify and map all metabolites onto metabolic pathways, to develop quantitative metabolic models for model organisms, and to relate organism metabolic pathways within the context of evolutionary metabolomics ^22^. To assist the experimental community in generating and gathering high-quality metabolomics data about biological models, an Implementation Study proposal called “MetabolHomes” has been submitted to ELIXIR as part of the present Metabolomics Use Case, which aims at providing users with a generic data model and the associated software tools for data management, visualisation, and annotation.

### Tools and standards registries

Since its inception, the Metabolomics community has developed a plethora of computational tools for data analysis ( https://goo.gl/Crf2Ye), as well as data and minimum information standards ( https://goo.gl/gouSQY). There is a general feeling that it is difficult for the average researcher to navigate both areas.

The suggestion here was to contribute to and improve long-term supported registries of tools and standards that help researchers decide which mature, well-tested tools and standards to use for which purpose. There is a need for more metabolomics resources to be appropriately included in Tools and Data Service Registry (tools), and the FAIRsharing registry (standards, databases, repositories and data polices), the two resources part of the ELIXIR platforms".

### Compound identifier mapping

In order to interpret the biological relevance of measured metabolites, their structures must be mapped to existing knowledge i.e., to pathways using a multi-omics data integration approach. Many metabolomics experiments measure a metabolite and characterise the structure with a retention time, one or more m/z values, or an NMR spectrum. Sometimes this characterisation can be linked to an identity of a specific chemical compound but often it can be only linked to a compound class. However, the tagged identity is commonly different from what is found in metabolism knowledge bases. In fact, different knowledge bases may have different focuses and representations of compounds. For example, one knowledge base may focus on the biological role of the metabolite, while another contains precise representations of the metabolites chemical structure and properties. Furthermore, knowledge bases are typically either too broad or too narrow in scope, and frequently not interoperable. Chemical structure mapping is therefore an important aspect to ensure interoperability between experimental and biochemical resources.

No generic solution currently exists, and people use either mapping based on expert knowledge ^23, 24^ or on equivalence based on the chemical structure, e.g. with the InChI string or key ^25, 26^. However, neither approach is well-suited for solving the issues around ambiguities in the characterisations of both the experimental side and the knowledge side. Theoretical solutions exist for linking these facts, such as scientific lenses ^27^, but these need to be extended to service the metabolomics research field.

In addition, pattern recognition analysis, such as Pavlidis template matching (Pavlidis and Noble, 2001) could further assist in identifying the biological role of the metabolite in the metabolic network and add to its chemical identification. Pavlidis template matching clusters a metabolite based on its concentration pattern with other metabolites of known identity or class in an experiment (or multiple experiments that are combined in a meta-analysis).

### Omics data integration

Metabolites function as both reactants and products of metabolic reactions. However, they also serve as regulatory molecules of proteins, affecting the structure and control of protein interaction and gene regulatory networks. This dual role of metabolites ensures that metabolomics is an integral aspect of systems biology research. 

There is a great need for standardised integrated multi-omic analyses for the comprehensive understanding of the cellular physiology with significant applications in biomedicine and all the spectrum of biotechnology. Thus, establishing standardized protocols of multi-omic (i.e. metabolomic, transcriptomic, proteomic, and interactomic) data representation, integration, visualization and interpretation is of great importance. Currently, metabolomics data can be integratively visualised with transcriptomics data. However, there is a lack of integrated omic databases for most model systems and it is not self-explanatory how a genomicist/proteomicist could integrate his/her data with metabolomics data that refers to the same biological system and *vice versa*. Additionally, there is a lack of harmonization of the experimental design, sample collection, handling and quenching protocols of metabolomics monitoring, which further increases the challenge of integrated omics analysis. The ELIXIR group proposed that these issues of integrated omic analysis and the standardization of metabolomic data interpretation in this context, could be tested and explored via comparison and analysis of a controllable reference biological system, such as a well-characterized human cell line. 

Genome scale metabolic networks and metabolic pathway databases contain information both on metabolites and their reactions with corresponding genes and proteins. Thus, these networks provide valuable context for simultaneous interpretation of metabolomics data and other omics data. However, mapping metabolites in these databases is a heavy workload (see above), since most of databases use specific identifiers for small molecules, where ideally chemical structure mapping should be applied. This is a particularly striking issue with genome-scale metabolic models, as these were initially built for constraint-based computational studies (flux balance analysis and related), where the chemical structure of small molecules plays no role. Because of this, no effort was put into those models to use proper small molecules identifiers, and instead only short and ambiguous names were used for small molecules, making mappings very difficult. Hence, omics data integration was not considered at all in their design and most of these databases (available in SBML format) do not provide standard metabolite identifiers. The MSI recommends a number of identifiers e.g. HDMB ID, ChEBI ID, CAS ID, and IUPAC Name, but only InChI and InChIKey encode the chemical structure themselves.

Recently, the Recon human genome-scale metabolic reconstruction network was enriched to incorporate InChIs, but more comprehensive chemical structure mapping is needed to capture all of the biological details. However, still most of the existing genome-scale metabolic reconstructions available for other organisms have not been enriched at this level. There is thus a strong need to coordinate with this community in order to facilitate the integration of metabolomics data in the context of these networks.

The problem of integrating metabolomics data into genome-scale metabolic models does not end in the community being able to map small molecules available there to proper identifiers, it only begins there. Classically, most established analysis methods used to simulate those models (Constraint-based analysis methods) are not prepared to use metabolite concentrations/abundances, as they only aim to balance incoming and outgoing reaction fluxes on each metabolite. So the use of metabolomics data in the context of these networks will present new challenges to the modelling community as well.

### Metabolite identification

Unlike genomics, the analytical Platforms used in metabolomics and lipidomics will not *per se* deliver a molecular identity, i.e. a specific chemical compound, but only the spectral characteristics. In untargeted metabolomics, metabolite identification remains the main bottleneck in data analysis and interpretation ^28^. The typical output is a spectrum containing (a large number of) features, which are characterised in NMR by the location and intensity of signals on a frequency axis, and in MS by *m/z* values (and possible additional information like retention time if coupled to a chromatographic system or drift times if coupled to ion mobility). In targeted metabolomics, data acquisition instrument parameters are tuned to detect (a list of) target compounds, thus making it possible to deliver tables of metabolite abundances, ideally with absolute quantification, for downstream biochemical interpretation. In recent years, both approaches have been improved towards each other, resulting in widely targeted metabolomics, covering hundreds of compounds ^29^.

Furthermore, computational tools for untargeted metabolomics methods have improved their ability to deliver metabolite annotation, albeit with varying levels of certainty ^30^. Concerted efforts in ELIXIR and the community can facilitate to improve the current situation, by removing the burden on developers to manually connect different tools into pipelines, and on experimentalists, by providing the tools and resources that give access to the knowledge required for biochemical interpretation of the data.

## Metabolomics Use Case in ELIXIR

After extensive discussions during the workshop, metabolite identification was identified by popular vote as the one area where:

A. A maximal impact of computational metabolomics and metabolomics data management could be aligned with other fields, in particular with the existing four ELIXIR Use Cases ( https://www.elixir-europe.org/use-cases).
B. Metabolomics community would benefit most
C. Can be exhaustively mapped onto the existing five ELIXIR Platforms.

## ELIXIR technical activities

ELIXIR technical activities are performed by ELIXIR Nodes and supported by the Hub. The Nodes run bioinformatics resources and services focusing on national priorities and contributing to a harmonised strategy across Europe. At the national level, an ELIXIR Node consists of research institutes within a member country, building on their national strengths. At the European level, ELIXIR’s activities are structured around Platforms and Use Cases, which bring resources and expertise together from both the ELIXIR Nodes and the basic unit of operation within ELIXIR. The ELIXIR Platforms are responsible for the implementation of the ELIXIR Scientific Programme, which is organised into five key areas: Data, Tools, Compute, Interoperability and Training.

The four Use Cases that currently represent four scientific communities are: Human Data, Rare Diseases, Marine Metagenomics and Plant Sciences. The Use Cases drive the work of the ELIXIR Platforms by describing their bioinformatics requirements. A close collaboration between the ELIXIR Use Cases and Platforms safeguards services developed by the ELIXIR Platforms would be fit for purpose.

Metabolomics activities are well represented within Europe and ELIXIR nodes. Following the establishment of national efforts such as the French MetaboHUB ^31^ and the Netherlands Metabolomic Centre, transnational efforts such as the FP7 coordination action COSMOS and the H2020 e-infrastructure PhenoMeNal followed. ELIXIR Greece has included a computational metabolomics and protein interactomics Use Case in the strategic planning for the national infrastructure. An ELIXIR Use Case on Metabolomics can have a positive impact on the community, strengthening collaboration and delivering a more harmonised strategy amongst service providers. Metabolomics aligns with the ELIXIR Platforms and Use Cases as well as other Omics themes represented and proposed in ELIXIR like Genomics and Proteomics. Though “metabolite identification” is not the only activity of interest within the ELIXIR Nodes, we proposed this Use Case as a starting point of common interest to catalyze the collaboration amongst ELIXIR Nodes and ELIXIR Platforms.

## Alignment with ELIXIR Platforms

The Metabolomics community in ELIXIR has vast expertise in the five areas represented by ELIXIR Platforms (Data, Tools, Interoperability, Compute and Training). Next, we summarise the current Platforms’ priorities, how the selected metabolomics Use Case aligns with the Platforms and a general alignment of metabolomics activities within Europe.

### 1. Data Platform

The Data Platform focuses on sustaining long term Europe’s life science data infrastructure by working on guidelines and indicators to improve data resources impact and long-term sustainability. Additionally, this platform aims to improve links between curated and non curated data resources and literature.

On the data side, metabolite identification requires a) the availability of high-quality curated resources for compound de-replication (the process of finding known chemical compounds in databases based on their spectroscopic and chromatographic fingerprints) as well as b) the establishment of workflows to push data on newly identified metabolites into the existing repositories. For a), the reference layer of the MetaboLights ^32^ database plays a crucial role and needs to be extended. The reference layer holds information about individual metabolites, their chemistry, their spectral data (MS, NMR), as well as their role in pathways and biological systems. New metabolites identified in studies deposited into MetaboLights ( http://www.ebi.ac.uk/metabolights/) are being curated by the MetaboLights team and added to the reference layer. In particular, characterization of the metabolome of biological models (e.g. organisms, tissues, biofluids, cell lines) is of major importance for the understanding of biochemical mechanisms, for the exploration of phenotype diversity and for the identification of new biomarkers. Due to the variety and the complexity of each biological system, gathering and curating knowledge about metabolomes can be best achieved by expert communities. In genomics, the GMOD project ( http://gmod.org/wiki/Main_Page) provides biological research communities with open-source software components for annotating and managing data about model organisms. Developing such data models and software tools in metabolomics to gather, analyse, and curate data will therefore be critical to produce high-quality knowledge about model metabolomes. The curated data (spectra, compounds, networks) and workflows will be of high value as input for the corresponding reference repositories and e-infrastructures (MetaboLights, ChEBI, MetExplore, Workflow4Metabolomics, PhenoMeNal).

Europe is a major provider of massive and high-quality metabolomics data. Large endeavors such as the MRC-NIHR National Phenome Centre ( http://www.imperial.ac.uk/phenome-centre), Phenome Centre Birmingham ( http://www.birmingham.ac.uk/research/activity/phenome-centre/index.aspx), the Netherlands Metabolomics Center ( http://www.metabolomicscentre.nl), and the French MetaboHUB ( http://www.metabohub.fr/home.html ^31^) infrastructure are producing data in key scientific and socio-economic areas, including the ELIXIR Use Cases ( https://www.elixir-europe.org/use-cases). Valorisation of this wealth of data requires annotation practices to be refined and new software tools to be developed to assist chemists in formatting, validating, referencing, and curating their annotations. All parties mentioned above have been engaging in the European GO-FAIR ( https://www.dtls.nl/go-fair/) initiative through their participation in PhenoMeNal, which has recently been co-organising the launch of a hub for FAIR metabolomics data in goFAIR. The FAIR data movement has gained considerable momentum in Europe, where FAIR ^33^ stands for data being Findable, Accessible, Interoperable and Reusable.

### 2. Tools Platform

The Tools Platform drives access and exploitation of bioinformatics research software by working closely with services and connectors. Additionally, this Platform aims to facilitate the discovery, benchmarking and interoperability of bioinformatics software by focusing on software development, best practices and on strategy for workflows and software containers.

Workflow management e-infrastructures such as Workflow4Metabolomics ( http://workflow4metabolomics.org/) ^34, 35^, PhenoMeNal, and Galaxy-M ^36^ are key European resources built on the Galaxy environment ^37^ that simultaneously address the two challenges of 1) high-performance, user-friendly, modular, and reproducible data analysis (needed by the experimental community), and 2) collaborative contributions from the bioinformatics community. Comprehensive workflows for preprocessing, statistical analysis, and annotation of data from liquid chromatography - MS (LC-MS), direct infusion MS (DIMS), gas chromatography - MS (GC-MS), and NMR technologies can be created, tailored, run, saved, shared, and publicly referenced with digital object identifiers ( http://workflow4metabolomics.org/referenced_W4M_histories). A recent questionnaire has shown a need to further develop such tools and workflows, as part Galaxy, that are well supported through community-based training, to further improve the standardisation and automation of data processing and analysis ^38^.

Standardization of compound annotation is critical for such Platforms i) to enable individual modules to communicate between each other and with external resources (e.g., repositories for raw data, mass spectra, compounds and metabolic networks) and ii) to deliver useful and FAIR data to the end-user. Conversely, new modules can be developed to integrate and harmonize annotations from complementary resources.

### 3. Interoperability Platform

The Interoperability Platform provides support to the discovery, integration and analysis of biological data organised in projects, centred around persistent identifiers, metadata and data standards for exchange and storage formats in addition to controlled vocabularies and linked data. This Platform facilitates work on the description of interoperability services and organises specialised BYOD (Bring Your Own Data) workshops with the aim to improve the FAIRness of data resources.

Experimental metabolomics data must be interoperable in order to facilitate integration with existing knowledge bases and other omics data. The collaborative development the ISA ^39^ framework for experimental metadata standards will help achieve such interoperability, as it has already embedded in the ELIXIR Plant Use Case. ISA will serve as bridging element with other omics applications and the FAIR sharing movement with this Metabolomics use case".

Interoperability can be realized by community accepted data standards and ontological molecule representations; a relevant list is available on FAIRsharing ( https://fairsharing.org/collection/H2020PhenomeandMetabolomeaNalysisPhenoMenalProject), a resource of the ELIXIR Interoperability Platform."

Persistent Identifiers for metabolites are a central need here, as is the more general chemical structure mapping problem (see above). The latter is a need that this metabolomics Use Case has in common with the Human Data, Rare Diseases, Marine Metagenomics and Plant Science Use Cases. The interoperability needs for metabolomics, however, extends beyond chemical structures: more standardized interoperability of experimental data, such as NMR spectra, is also required. Introduction of the SPLASH ^40^ for NMR would benefit the other Use Cases too.

### 4. Compute Platform

The Compute Platform is devoted to the compute, transfer, storage, authentication and authorization related to biological data relying on services provided by ELIXIR Nodes and other e-infrastructures.

With PhenoMeNal, Europe now has at least one major initiative to support computing with large-scale metabolomics data. The PhenoMeNal e-infrastructure enables researchers to deploy and test metabolomics workflows in public clouds (Amazon EC2, Google Compute Platform) or local, in-house OpenStack environments in cases where sensitive data cannot leave the institution. It also provides a number of commonly used workflows for metabolomics that include the eventual identification of metabolites in metabolomics experiments and the mapping onto biological pathways. PhenoMeNal unites major metabolomics laboratories across Europe and forms an essential component for our next steps to launch a European infrastructure for metabolomics service laboratories.

### 5. Training Platform

This Platform aims to increase the professional skills for managing and exploiting data. The training activities focus on researchers, trainers and service providers, but also include e-learning, the discovery of training materials and measuring the impact of training.

The need for further development of training programmes in metabolomics across Europe is well recognised, including training in metabolite identification ^41^. Over the last few years multiple training courses have been established, somewhat ad hoc, both in relatively large training centres as well as individual laboratories that specialise in a particular aspect of metabolomics. Currently, there is a critical need to improve the coordination between these training courses and initiatives, and to ensure that all stakeholders across Europe and beyond (e.g. NIH training centres in USA and Metabolomics Australia) are able to readily access courses, from introductory to advanced, including online and face to face. This is one of the objectives of the newly formed European Metabolomics Training Coordination Group (EmTraG, http://www.emtrag.eu), led initially by a team in ELIXIR-UK with support from several other ELIXIR Nodes and the ELIXIR Training Platform.

Specifically in the context of metabolite identification, several introductory training courses teach the basics of metabolite annotation and identification. In addition the Birmingham Metabolomics Training Centre (BMTC), an ELIXIR-UK training resource, runs a course “Metabolite identification with the Q Exactive and LTQ Orbitrap” in partnership with Thermo Scientific. Formalising a Metabolomics Use Case within ELIXIR could enable the expansion of the delivery of such courses, for example through the EmTraG network. Training partnerships with instrument vendors can be extremely valuable, increasing the quality of the training material and facilities, as also has been achieved by Waters Corporation partnering with the Imperial International Phenome Training Centre and the BMTC.

Training in metabolite identification requires materials and case studies related to both data acquisition and bioinformatic analysis of the acquired data and a multi-disciplinary training team of analytical chemists and bioinformaticians to deliver courses. The provision of courses currently focuses on hands-on training at training centres, as described above at the BMTC, and which typically can train 6–12 scientists per course. However, in the growing discipline of metabolomics, there is a requirement to provide training to larger numbers that is only achievable through online training resources. The matching of trainee learning objectives to the type of course provided is key and recent examples of online courses have demonstrated their power in delivering Massive Open Online Courses (MOOCs) or more specialised Small Private Online Courses (SPOCs). At BMTC, the introductory course on metabolomics MOOC has been used by greater than 3000 active learners and the first SPOC focussed on data processing and analysis in metabolomics was completed by more than 50 people. However, courses for greater levels of hands-on training in the laboratory are focused on training in the laboratory; through the use of video media we can envisage some courses operating via online resources.

For training purposes, the Galaxy framework has also shown that it could be an efficient Platform to explain tools, parameters and workflows to life scientists without any skills in scripting (R, Bash, Python). The trainees can focus on their scientific questions regardless the technical aspects and programming language barrier. Since 2014, the Workflow4Metabolomics group (ELIXIR-FR, MetaboHUB) have conducted three sessions of one week based on their Galaxy instance. For those who wish to use the command line after the training session, the bridge is easy since the parameters within Galaxy are mapped exactly on the native software.

ELIXIR training modules can be classified into three types of trainers:

1) Life Scientist (TrR): “Bring Your Own Data” training addressed to the experimentental community optimally promote good practices for using software and critically interpreting the results, and provide feedback about specific training needs. As an example, during the Workflow4Experimenters (ELIXIR-FR, MetaboHUB) one-week courses (W4E) ( http://workflow4metabolomics.org/events), participants learn to analyze their own MS or NMR datasets by using the Workflow4Metabolomics Platform. Morning sessions are dedicated to methodology and tools and afternoon sessions are devoted to tutoring. Such training offers unique opportunities to discuss the designs, methods, and tools for comprehensive and rigorous data preprocessing, statistical analysis, and annotation.

2) Communities of Developers (TrD): to enrich the tools and compute Platforms based on good practices guidance. As an example, the ELIXIR-EXCELERATE and IFB (ELIXIR-FR) European Galaxy Developer Workshop (EGDW, https://www.elixir-europe.org/events/elixir-excelerate-and-ifb-european-galaxy-developer-workshop), hosted in Strasbourg, aimed to teach the best-practices about the tool integration and advanced features like the Galaxy API, visualisation and administration (high performance computing (HPC), Docker).

3) Trainers expert (TrT): Train the trainers program with the best strategies in Learning principles and didactic strategies for delivering bioinformatics in the most effective way

## Alignment with ELIXIR Use Cases

The ELIXIR Platforms are currently complemented by four Use Cases across four scientific communities:

1. The Human Data Use Case for long-term strategies for managing and accessing sensitive human data.
2. The Rare Disease Use Case for development of new therapies for rare diseases.
3. The Marine Metagenomics Use Case works towards a sustainable metagenomics infrastructure to support research and innovation in the marine domain.
4. Plant Science Use Case for infrastructure development for genotype-phenotype analysis of crop and tree species.

As one of the core omics technologies, metabolomics forms an important component in all of the science-driven Use Cases established so far in ELIXIR. In particular, for the human data Use Case, the PhenoMeNal e-infrastructure is very relevant as is working to develop cloud-based resources for computing with big clinical metabolomics data. Significant synergies between the privacy and ethics work package in PhenoMeNal and the parties working on the human data Use Case are obvious.

As one of the best established molecular phenotypes, metabolomics is already widely used for the genotype-phenotype analysis of crops and in tree species, providing an immediate interplay with the Plant Science Use Case which is also supported by the common of use of the ISA experimental metadata framework.

A rich corpus of work exists on the metabolomics of marine organisms where communities of, for example, marine sponges and marine microorganisms produce pharmacologically active polyketides with diverse chemical structures, which are investigated based on genomic and macroeconomic data of these communities.

In all those application scenarios, the mapping of spectral features in metabolomics data to identified chemical compounds (metabolites) and thereby to molecular pathways is crucial for the understanding of the biochemistry underlying the Use Case in question.

Following the formation of national infrastructures for metabolomics, for example with the French infrastructure in Metabolomics and Fluxomics MetaboHUB and the Netherlands Metabolomics Centre Foundation (NMC), the COSMOS initiative ^7^ for the coordination of standards in metabolomics provided the first coordination action between all relevant efforts in metabolomics in Europe. Leading to the establishment of the worldwide metabolomeXchange network, COSMOS paved the way for PhenoMeNal. As a core organiser of the launch of the node for FAIR data ^33^ in metabolomics as part of the goFAIR initiative, the PhenoMeNal consortium made another necessary step to establish itself as an authority for metabolomics data ELIXIR Greece has included a computational metabolomics and protein interactomics Use Case, including the formation of standardized integrated metabolomic and proteomic databases and the evolvement of tools for combined metabolic and protein network analysis, in the strategic planning of its national infrastructure management and processing for the European Open Science Cloud (EOSC).

Significant synergies can been leveraged with the suggested sister Use Case for proteomics ^42^. The metabolomics and proteomics communities have been extensively interacting on the data standards and formats side, where metabolomics has been able to adapt and adopt work that has been started by the proteomics community. Based on these preparatory steps, our proposal to establish metabolomics as a Use Case in ELIXIR is a logical progression.
