# Supplementary material for: The future of metabolomics in ELIXIR
Source: F1000Res. 2017 Oct 30;6:ELIXIR-1649. Originally published 2017 Sep 6. [Version 2] doi: 10.12688/f1000research.12342.2 (PMC5627583; doi:10.12688/f1000research.12342.2)
Supplement: Peer review history

# Peer review: Referee response for version 2

**DOI:** 10.5256/f1000research.13993.r27416

**Published:** 2017-11-28

**Authors:** Carl Brunius

**Affiliations:** 1 Chalmers University of Technology, Gothenburg, Sweden

I support the decision to accept the manuscript and I have no comments to add.

I have read this submission. I believe that I have an appropriate level of expertise to confirm that it is of an acceptable scientific standard.

# Peer review: Referee response for version 1

**DOI:** 10.5256/f1000research.13363.r25890

**Published:** 2017-09-29

**Authors:** Emmanuel Mikros

**Affiliations:** 1 Department of Pharmacy, National and Kapodistrian University of Athens, Athens, Greece

The manuscript presents an opinion paper concerning the outcomes of the workshop that took place within the context of the strategic planning of ELIXIR and PhenoMeNal consortia. The article describes the actual level and the diversity of efforts within the European Metabolomics community. It represents main bottlenecks in reporting QA/QC, the metabolite identification, and the lack of comprehensive and coordinated metabolite databases. The authors describe their plans how to address challenges in metabolomics mainly within the ELIXIR platforms. The paper is undoubtedly, informative for the major part of common efforts of the European Metabolomics Community. It is mainly focusing on ELIXIR plans yet the intentions of the authors are to inform the community on this issue and this is clearly reflected in the title.

 This reviewer feels that the complexity of the non human metabolome (plant, microbe etc) is not appropriately addressed and the relative databases of secondary metabolites existing worldwide should be more seriously considered.

 Comments:

 Some corrections should be considered before indexing.

- In page 5 the statement “For NMR, a few and sparsely populated repositories provide raw data for individual metabolites, such as the metabolomics collection in BioMagResBank” is somehow contradictory with the fact that HMDB is mentioned just few sentences later and MetaboLights is also mentioned in a completely different context in another paragraph.
- The sentence “ELIXIR Greece has included a computational metabolomics and protein interactomics Use Case” is repeated twice in page 7 and page 10
- In page 6 the reference Pavlidis and Noble 2001 appears without any relevant number and is not mentioned in the references section

I have read this submission. I believe that I have an appropriate level of expertise to confirm that it is of an acceptable scientific standard.

# Peer review: Referee response for version 1

**DOI:** 10.5256/f1000research.13363.r25762

**Published:** 2017-09-18

**Authors:** Carl Brunius

**Affiliations:** 1 Chalmers University of Technology, Gothenburg, Sweden

General comments:

 I have answered “partly” to some of the questions above, since this article constitutes a condensation of a workshop/meeting and as such contains opinions, not necessarily easily backed with either results or literature. However, it should be noted that the authors have done a remarkable job of justifying opinions and claims as far as possible.

 The authors have submitted a manuscript which to a large degree gives an accurate representation of challenges and bottlenecks in metabolomics along the pipeline from data capture down to biological interpretation and data integration. The identification of metabolite identification as the single major challenge reflects well the discussions in the metabolomics community. The article places emphasis on the maturity of metabolomics and expertly points out the need for integration of platforms and infrastructure, with an emphasis on European research.

 The manuscript promotes a phenome-oriented view on metabolomics, with emphasis on bioinformatics of known/identified compounds and permeating a systems biology view at the expense of the more explorative side of untargeted approaches. This is understandable from the perspective of Elixir and PhenoMeNal, but somewhat misrepresentative of the larger metabolomics community, which obviously hasn’t yet matured to the stages of other (older) omics disciplines due to e.g. technical heterogeneity in platforms and difficulties in identification. On the other hand, these potentially different perspectives converge in the emphasis on metabolite identification.

 The manuscript is furthermore likely difficult to penetrate for those readers that may not be fully up to date on European and international efforts for streamlining bioinformatics infrastructures. A more thorough introduction would help lower that threshold.

 More detailed comments:

 The article pre-supposes knowledge from the reader on organisations, infrastuctures and current research political agendas on European level, as well as what similar trajectories can be observed outside of Europe. A presentation of past efforts in streamlining standards and needs for international organisations, as well as a more thorough introduction on Elixir, PhenoMeNal and other initiatives would have been in order to help the less initiated reader to follow the article. These background issues should be more thoroughly explained, broadened and put into relevant context to reduce the feeling of this being an Elixir-internal lobbying paper. E.g.

- Consider in relation to this aspect that: “The objective of the meeting was to identify the principal challenges within this field and prioritise actions, in particular those within the scope and mission of ELIXIR.” AND the article title: “The future of metabolomics in ELIXIR”
- The authors state that: “ELIXIR coordinates bioinformatics resources across its member states and help researchers to find, analyse, and exchange biological data. It is a distributed infrastructure with a single Hub based in Hinxton, United Kingdom, and an increasing number of Nodes located throughout Europe. As of July 2017, ELIXIR has 20 national Nodes, with European Bioinformatics Institute (EMBL-EBI; co-located with the Hub), working as a separate Node.” This description, however, hardly describes Elixir from a functional point of view.
- Moreover, considering that: “This opinion article is a call for support for a new ELIXIR metabolomics Use Case” there is surprisingly little information on the Elixir use cases in the background section. What are the aims, objectives and functionalities of the use cases and how do they relate to the core challenges in the metabolomics community? Overlaps between use cases and challenges are addressed later in the article, but for something this important, this should have been addressed in the introduction. 

The challenges/bottlenecks in metabolomics have been expertly identified. However, these challenges are not exclusive to Elixir, but represents challenges to the entire metabolomics community.

Correction: 2. “Genome-based metabolic reconstruction databases: These databases are built based on genome annotation, mostly of enzymes and their association to known reactions, and thus may not cover the entire metabolome since some genes that are enzymes have no enzymatic function associated”

 The section on current standards and techniques for multiOMICs integration is sketchy: “Currently, metabolomics data can be integratively visualised with transcriptomics data.”. There are several interesting and promising initiatives for data integration extending well beyond visual integration with transcriptomics as the authors are well aware. There is, however, a lack of validated tools. Especially for supervised data analysis of integrated data (data integration isn’t limited to systems biology through pathway analysis, but is also highly relevant from a biostatistical point of view). But I also agree that there is “a lack of integrated omic databases”, although I believe that there are several crucial aspects of data integration that need to be addressed well before we go into database issues. The systems biology approach will furthermore not be advisable in an untargeted metabolomics setting: Where complementarity in biological information from the different omics layers may be utilised for predictive modelling of e.g. disease pathophysiology of risk modelling before compound identification (and may, in fact even be used as a strategy for compound identification through e.g. Bayesian network modelling).

 Regarding metabolite identification, the authors state: “Concerted efforts in ELIXIR and the community…” The order should probably be the inverse.

Correction: “The Data Platform focuses on sustaining long term Europe’s life science data infrastructure”

 A reflection on the “Data Platform” section is that many researchers seem to have perceived reporting formats for the MetaboLights service as somewhat of a hurdle (as are most “general” issue added value tasks not corresponding to the core task at hand for most funded research (e.g. identifying predictive biomarkers of a specific disease). Whereas I have seen several BYOD initiatives for actual analysis of data, I still haven’t seen BYOD approaches would metadata handling. Maybe these exist – From a user-friendliness perspective it would be interesting to see how this hurdle is being tackled. It would also be interesting to have survey results on perceived bottlenecks in metabolights (and similar repositories) if such exist, which could potentially focus efforts into FAIR reporting.

I have read this submission. I believe that I have an appropriate level of expertise to confirm that it is of an acceptable scientific standard, however I have significant reservations, as outlined above.

# Peer review: Referee response for version 1

**DOI:** 10.5256/f1000research.13363.r25758

**Published:** 2017-09-15

**Authors:** Charles F. Burant

**Affiliations:** 1 Department of Nutritional Sciences, University of Michigan School of Public Health, Ann Arbor, MI, USA; 2 Department of Internal Medicine, University of Michigan Medical School, Ann Arbor, MI, USA

This article is a nice summary of a meeting of European scientists who have a range of interests in metabolomics-based science.  The review provides a summary of the main areas that the investigators considered critical to move move both the technological aspects of the work forward, with securing the identification of 'unknown' metabolites found in biological samples, as the primary need.  The group also identified various aspects of data science, data science and bioinformatics as other high priority needs.

 I found the review to be detailed, informative and well referenced. The review is unabashedly Eurocentric, appropriately so given that it was generated with the goal of highlighing European efforts.  Thus, it sometimes reads as an advert for ELIXIR, but that does not distract measurably from the fine recitation of the opinions of the participants.

I have read this submission. I believe that I have an appropriate level of expertise to confirm that it is of an acceptable scientific standard.
